# Maternal Lipid Concentrations during Early Pregnancy and Eating Behaviour and Energy Intake in the Offspring

**DOI:** 10.3390/nu10081026

**Published:** 2018-08-06

**Authors:** Anna M. Dieberger, Susanne R. de Rooij, Aniko Korosi, Tanja G. M. Vrijkotte

**Affiliations:** 1 Department of Public Health—Amsterdam Public Health Research Institute, Academic Medical Centre, University of Amsterdam, Meibergdreef 9, 1105 AZ Amsterdam, The Netherlands; anna.dieberger@gmail.com (A.M.D.); s.r.derooij@amc.uva.nl (S.R.d.R.); 2 Department of Clinical Epidemiology, Biostatistics and Bioinformatics—Amsterdam Public Health Research Institute, Academic Medical Centre, University of Amsterdam, Meibergdreef 9, 1105 AZ Amsterdam, The Netherlands; 3 Swammerdam Institute for Life Sciences, Centre for Neuroscience, University of Amsterdam, Science Park 904, 1098 XH Amsterdam, The Netherlands; a.korosi@uva.nl

**Keywords:** CEBQ, eating behaviour, energy intake, foetal programming, FFQ, lipids, pregnancy

## Abstract

Worldwide, childhood obesity is rapidly increasing, making it a pressing public health issue. Obesity is strongly linked to eating behaviour and energy intake but little is known about their prenatal determinants. In an exploratory study of data collected within the Amsterdam Born Children and their Development (ABCD) study, we hypothesized that intra-uterine exposure to increased lipids is associated with adverse eating behaviour and increased energy intake in the offspring at age 5. During early gestation, a non-fasting blood sample was taken from 1463 non-diabetic Dutch women, including: total cholesterol (TC), triglycerides (TG), free fatty acids (FFA), Apolipoprotein A1 (ApoA1) and Apolipoprotein B (ApoB). Eating behaviour, measured using the Children’s Eating Behaviour Questionnaire, included food approaching (enjoyment of food, food responsiveness) and food avoidant behaviour (satiety responsiveness, slowness of eating). Energy intake (total energy, fat and carbohydrate intake) was measured using a validated food frequency questionnaire. Associations were analysed using multivariable linear regression. Increased maternal TC concentrations were associated with lower enjoyment of food, higher satiety responsiveness and increased slowness of eating, as well as decreased kcal and fat intake in the offspring. Elevated ApoA1 was associated with increased slowness of eating, lower enjoyment of food and lower kcal, fat and carbohydrate intake. ApoB was positively associated with satiety responsiveness and slowness of eating. Higher TG concentrations were associated with higher food responsiveness. Maternal FFA did not show significant associations. Findings demonstrated that the maternal prenatal lipid profile was associated with offspring’s eating behaviour and energy intake, although not always in the hypothesized direction.

## 1. Introduction

The global prevalence of childhood overweight and obesity is steadily on the rise, showing an increase in obesity from 0.7% to 5.6% in girls and from 0.9% to 7.8% in boys worldwide between 1975 and 2016 [1]. Obesity is related to numerous comorbidities such as diabetes, cardiovascular diseases and cancer, making it a worldwide health hazard and a growing burden on health care and related costs [2,3]. Childhood obesity has moved more and more into focus in recent years, as obesity in children and its associated risks often persist into adulthood [4].

Obesity is caused by a long lasting positive energy balance and its rise in recent years can be explained by environmental changes, entailing an increase in sedentary lifestyles and energy dense food availability [5,6]. However, not every individual is turning obese, despite being exposed to the same obesogenic environment [6]. This could be due to a variation in perceptiveness to these environmental exposures, expressed as differences in eating behaviour [6]. Eating behaviour regulates the selection and preference of foods as well as managing timing and quantity of food intake [7]. It is steered largely by the central nervous system [5] and its development is influenced by genetic as well as environmental factors [6]. The theory of behavioural susceptibility of obesity states that adverse traits of eating behaviour, namely decreased food avoidant behaviour and increased food approaching behaviour, are associated with increased energy intake, a positive energy balance and consequently obesity [6,8]. Several observational studies have confirmed an association between adverse eating behaviour and energy intake [9], weight [10] and obesity in children [11].

Emerging evidence shows that the development of eating behaviour can already be influenced as early as in utero [12]. For instance, several animal studies conducted with pregnant rats and mice have confirmed that maternal obesity and over-nutrition with a high-fat, high sugar diet compared to a normal diet leads to significantly increased food intake and perturbed satiety in the offspring, before the onset of obesity [13,14,15]. Animal studies focused on the development of eating behaviour showed that maternal over-nutrition leads to a reprogramming of the expression of orexigenic and anorexigenic neuropeptides such as AgRP and POMC in the offspring’s hypothalamus [15,16], which is the most important neural structure responsible for long-term energy homeostasis [5] and which is already developed in utero [17]. Although there is little evidence from human studies, results from the Dutch famine birth cohort study demonstrated that undernutrition during the first trimester of gestation was associated with an increased preference for fatty foods in later life, suggesting that eating behaviour can indeed be influenced by early life conditions [18].

The question remains which elements of the maternal diet are involved in the programming of offspring’s eating behaviour. Research has shown that the maternal prenatal lipid profile (including cholesterol, triglycerides and free fatty acids) is a strong determinant for the foetal environment [19,20] and that the lipid profile is positively associated with maternal fat intake in pregnancy [21]. Furthermore, we have recently shown that maternal lipid concentrations, such as total cholesterol, triglycerides, apolipoprotein B and free fatty acids, are positively associated with the offspring’s body composition at the age of five [22]. As eating behaviour and body composition are strongly associated, we hypothesized that an altered maternal lipid profile could influence the offspring’s eating behaviour and energy intake. Up until now, no human studies investigating this association, exist. Therefore, we set up an exploratory study aiming to investigate the association between the maternal lipid profile in early pregnancy and eating behaviour and energy intake of the offspring at the age of five.

## 2. Subjects and Methods

### 2.1. Study Design

This study is part of the Amsterdam Born Children and their Development (ABCD) birth cohort study. The ABCD study was designed to follow a community-based cohort from pregnancy to adulthood, examining parental factors and their influence on the child’s health and development [23,24].

### 2.2. Study Population

All pregnant women living in Amsterdam between January 2003 and March 2004 were invited to participate in the ABCD study during their first prenatal appointment with their obstetric caregiver. In total, 8266 responded by filling out an extensive questionnaire including general personal information, lifestyle habits and ethnic background (67% response rate) [24]. Of all participants, 4387 (53%) agreed to also participate in the biomarker study and thereby agreed to take an extra sample of blood during early pregnancy, in addition to the standard screening blood sample and were therefore eligible for this study [24].

**Figure 1:**
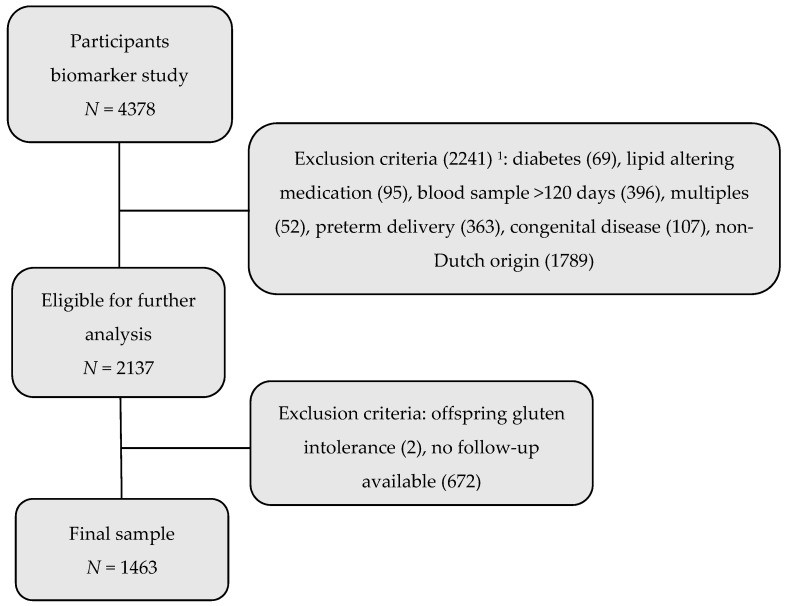
Flowchart presenting exclusion criteria and study population. ^1^ Numbers sum to more than 2241, as sometimes more than one exclusion criterion was present.

On the participants of the biomarker study, exclusion criteria were operated, which can be seen in Figure 1. All participants of non-Dutch origin were in the first instance excluded, because perceptions of eating behaviour and energy intake differ between ethnic groups as a consequence of variety in cultural backgrounds. While measurement tools for eating behaviour have been validated in different ethnic groups [11,25], doubts concerning validity in low-income or ethnically different cohorts remain [26,27]. Therefore, to reduce heterogeneity, only Dutch participants were included. Further exclusion criteria entailed women taking the blood sample during late pregnancy (>120 days; *n* = 396), women carrying multiples (*n* = 52), children with severe congenital abnormalities (*n* = 107) or who were born preterm (*n* = 363). Also, mothers having pre-pregnancy or gestational diabetes (*n* = 69), or those taking lipid-altering medication (antiepileptic drugs, steroids, insulin, antidepressants, thyroid hormones, sleep medication) (*n* = 95) were excluded. No participants were using antilipidemic drugs [24].

In 2008 and 2009, when the child was aged 5 to 6 years old, all participants were again asked to fill out a questionnaire to measure energy intake and eating behaviour in the offspring. Participants without follow-up information concerning energy intake and eating behaviour of the offspring were excluded (*n* = 672), as well as children with severe nutritional restrictions such as gluten intolerance (*n* = 2). This resulted in 1463 women and their singleton term offspring in the final study sample.

The study was approved by the medical ethics review committees of the participating hospitals and the Registration Committee of the Municipality of Amsterdam [24]. Written informed consent was provided by the mothers for themselves and their children [23,24,28].

### 2.3. Data Collection

From each woman, a non-fasting blood sample was taken, in addition to the standard blood sample at the first prenatal check-up during early pregnancy (mean; 12.7 weeks ± 1.8 SD). The blood was sampled in a 9-mL Vacuette (Greiner BV, Alphen aan de Rijn, The Netherlands) to prepare serum. In the serum, the lipid profile was determined, including total cholesterol (TC), triglycerides (TG), free fatty acids (FFA), apolipoprotein A1 (ApoA1) and apolipoprotein B (ApoB). For the assessment of TC and TG, a Hitachi 912 analyser (Roche Diagnostics, Mannheim, Germany) was used. TC was determined through the cholesterol oxidase PAP method, TG through the glycerol-3-phospate oxidase PAP method. The interassay coefficients of variation were 1.0% and respectively 2.3% for TC and TG. FFA were determined using a combination of enzymatic and spectrophotometry/colorimetry (reagent by Wako Pure Chemical Industries Ltd., Osaka, Japan). ApoA1 and ApoB were assessed using a turbidimetric technique. Both techniques were performed on an Abbott Architect CI8200 (Abbott Laboratories, Limited., Saint-Laurent, QC, Canada). The interassay coefficient of variation was 3.4% for FFA, 1.6% for ApoA1 and 1.6% for ApoB. Total cholesterol, triglycerides and FFA are presented in mmol/L, ApoA1 and ApoB in g/L [22].

Information on baseline characteristics was obtained from the pregnancy questionnaire and supplemented with information from obstetric files [28]. This included maternal height and pre-pregnancy weight, which were used to calculate maternal pre-pregnancy BMI in kg/m^2^, ethnicity (defined as country of birth of the woman’s mother), alcohol use (yes, no) and smoking during pregnancy (yes, no), parity, maternal educational status (measured as years of education after primary school) as an indicator of socio-economic status, prenatal anxiety (measured as a State-Trait Anxiety Inventory (STAI) questionnaire score [29]) and disease and medication status. Data on birth weight (grams), gestational age (weeks) and sex of the offspring as well as congenital abnormalities were acquired by combining information from questionnaires, youth health centres and the perinatal registry [22]. Regular weight and height measurements of the offspring during the first year of life were performed by trained nurses in national youth health centres. Based on this information, standard deviation scores (SDS) for infant weight were calculated [30]. Accelerated infant growth was defined as a positive change in SDS of the child’s weight of 0.67 or more between the age of approximately four weeks and six months [31]. Information on duration of exclusive breastfeeding (none, <1 month, 1–3 months, 3–6 months, >6 months), child’s medication use and diseases at five years of age (i.e., gluten intolerance) was acquired through parent-report questionnaires performed at several time points and was again combined with information from youth health care centre records [32]. Offspring height and weight at the age of five were measured at a health check, performed by trained research assistants of the study and BMI (kg/m^2^) was calculated [22]. Offspring BMI was categorized into underweight, normal weight and overweight, according to age and sex specific Dutch references for descriptive purposes [33].

The two outcomes of this study were eating behaviour and energy intake in the offspring and were ascertained when the child was aged 5 to 6 years old. To measure eating behaviour, a children’s eating behaviour questionnaire (CEBQ) was conducted. The CEBQ is a validated parent-report questionnaire, designed to measure eating behaviour related to obesity risk in young children [34]. The 35-item questionnaire consists of eight subscales, of which four subscales measure food approaching behaviours and four subscales measure food avoidant behaviours [35].

In the current study, four subscales were measured; enjoyment of food (EF) and food responsiveness (FR) as food approaching behaviours and satiety responsiveness (SR) and slowness of eating (SE) to measure food avoidant behaviour. EF reflects the child’s general interest in eating, while FR assesses the response to external food cues. SR assesses the ability to react to internal satiety cues and reduce food intake accordingly. SE reflects on a child’s ability to slow down food intake at the end of a meal [9,34]. Adverse eating behaviour can be seen as low scores in the latter two categories and high scores in EF and ER and are all associated with increased food intake [9] and higher BMI in children [10].

Each subscale is composed of three to six items, which is scored on a 1–4 point Likert scale (definitely not true–not true–true–definitely true). For each subscale, a mean score was calculated. These four mean subscale scores (EF, FR, SR, SE) were used to measure the outcome eating behaviour as continuous variables. Cronbach’s Alpha’s of the four subscales ranged between 0.79 and 0.80.

Energy intake was measured using a validated parent-report food frequency questionnaire (FFQ) for 4 to 6-year-old children [36]. The FFQ consisted of 71 food items including questions on portion sizes and frequency of consumption. From this, total daily kcal intake (kcal per day), fat and carbohydrate intake (grams per day) were calculated, using the Netherlands Food Composition Table NEVO 2001 [36]. The calculated daily kcal, fat and carbohydrate intake were then used as continuous outcome measures indicating offspring’s energy intake.

### 2.4. Statistical Analysis

Maternal blood lipid concentrations (TC, TG, FFA, ApoA1, ApoB) were interpolated for mean gestational age at blood sampling (90 days), using regression analysis, as lipid values normally increase over the course of pregnancy [21,37]. Descriptive statistics were used to display the study population’s baseline characteristics and mean values of outcome measures. Non-response analysis was performed comparing all included participants (*n* = 1463) to those of Dutch origin who gave a blood sample and complied with all inclusion criteria but did not provide follow-up information on the offspring’s eating behaviour (*n* = 672). Differences of baseline characteristics between the groups were calculated using t-tests and analysis of variance. Pearson correlations were calculated for EF, FR, SR, SE, kcal, fat and carbohydrate intake and offspring’s BMI to test whether these factors correlated in the expected direction of the theory of behavioural susceptibility of obesity.

The associations between maternal lipids and eating behaviour and maternal lipids and food intake were analysed using multiple linear regressions. All regression models satisfied assumptions to justify the use of linear regression analysis. Crude analyses were followed by multivariable analyses that were adjusted for the following a priori stated confounders: maternal educational level (years of education after primary school; continuous), pre-pregnancy BMI (kg/m^2^; continuous), smoking during pregnancy (yes, no), parity (primiparous, multiparous), maternal prenatal anxiety (continuous) and the offspring’s sex. We additionally adjusted for postnatal factors (duration of exclusive breastfeeding (none, <1 month, 1–3 months, 3–6 months, >6 months), accelerated postnatal growth (yes, no) and maternal weight gain since pregnancy (kg; continuous) as indicators for the child’s family environment. These confounders were chosen based on a directed acyclic graph which was made based on the theoretical background and literature. Some literature shows differences in the effects of foetal programming on several health outcomes depending on the offspring’s sex [13,38]. Therefore, effect modification by child’s sex was tested by creating an interaction term between the different lipids and the offspring’s sex and adding it to the multivariable regression analysis. If the added interaction coefficient had a *p*-value of <0.05, effect modification was assumed and results were stratified accordingly.

A secondary analysis was performed, including all ethnicities (*n* = 2044), to see whether the results were representative for the whole population.

Missing values of covariates (maximum percentage of missing values: 19.5%) were imputed by multiple imputations (20 datasets). The relatively high percentage of 19.5% missing values was found in the variable describing accelerated growth. This can be explained as for this variable several measurements per participants were summarized, resulting in a higher overall percentage of missing values. All other imputed variables had missing values between 0.1–2.9%. A *p*-value of <0.05 was deemed statistically significant. As the current study has an explorative character, correction for multiple testing was not applied, to increase chances of discovering possible effects. All analyses were performed using SPSS version 24.0 (SPSS Inc., Chicago, IL, USA).

## 3. Results

### 3.1. Study Population

In Table 1, baseline characteristics of all participants and their offspring (*n* = 1463) are presented by maternal lipid concentrations. The participating mothers had a mean age of 32.3 (SD 3.7) years during pregnancy, received on average 10.8 (2.9) years of education after primary school and had a mean pre-pregnancy BMI of 22.6 (3.5) m^2^/kg. The majority (59.7%) were nulliparous, non-smokers (92.0%) and did not consume alcohol during pregnancy (66.9%). Maternal education and pre-pregnancy BMI were significantly associated with concentrations of most lipids. All other associations with lipid concentrations are presented in Table 1.

Compared to mothers in the non-response group, mothers in the included group were significantly older (*p* < 0.001), higher educated (*p* < 0.001) and were more likely to consume alcohol in pregnancy (*p* = 0.010). The two groups did not significantly differ in smoking during pregnancy, parity and pre-pregnancy BMI (see supplementary Table S1).

Mean scores for CEBQ and food intake are presented in Table 2. The daily mean kcal intake of the offspring at 5 years was 1508, with a mean fat intake of 52 g and mean carbohydrate intake of 192 g per day. Mean scores for EF, FR, SR and SE were 2.56, 1.88, 2.32 and 2.41 respectively.

The different CEBQ subscales were all significantly correlated with offspring’s food intake and BMI (see Table 3). EF and FR were positively associated with kcal, fat and carbohydrate intake, while SR and SE were negatively correlated with food intake. EF and FR were positively and SR and SE were negatively associated with offspring’s BMI (*p* < 0.001). Offspring’s BMI and food intake were not significantly correlated.

### 3.2. Eating Behaviour

CEBQ scores were also compared according to baseline (see supplementary Table S2). The offspring’s EF scores were lower in mothers with pre-pregnancy underweight and higher in offspring with a birth weight of 4000 g or more. FR was significantly higher in offspring of young mothers, those with lower education, mothers with lower prenatal anxiety and offspring with accelerated postnatal growth. Offspring’s FR scores were lower in mothers with pre-pregnancy underweight. SR was significantly increased in offspring of mothers that were obese before pregnancy. SR and SE scores were lower in male offspring and in those with a high birth weight (>4000 g).

In Table 4, associations between the prenatal maternal lipid profile and the offspring’s eating behaviour at age five are presented. After adjustments, higher maternal TC concentrations were significantly associated with lower EF (β: −0.037; 95% CI: −0.067, −0.007) and higher SR (0.052; 0.021, 0.083) and SE (0.048; 0.012, 0.084) in the offspring. Higher ApoA1 was associated with lower EF (−0.128; −0.250, −0.005) and higher SE scores (0.162; 0.014, 0.309). ApoB was positively associated with SR (0.185; 0.027, 0.342) and SE scores (0.196; 0.011, 0.382). Higher maternal TG concentrations were associated with higher FR in the offspring (0.076; 0.023, 0.129). Maternal prenatal FFA were not associated with eating behaviour in the offspring.

### 3.3. Energy Intake

Table 5 presents associations between maternal lipid concentrations and food intake of the offspring. After adjustments, increased TC concentrations were associated with lower total energy intake (−26.33 kcal/day; −48.42, −4.24) and fat intake (−1.38 g/day; −2.36, −0.40). Higher ApoA1 concentrations were associated with lower kcal (−185.99; −271.87, −100.10), fat (−6.86 g/day; −10.66, −3.06) and carbohydrate intake (−23.55 g/day; −35.11, −11.99) in the offspring. ApoB, TG and FFA concentrations were not associated with offspring’s food intake.

### 3.4. Sex Specific Outcomes

Offspring’s sex did not act as an effect modifier in any model, except for the association between maternal FFA and offspring’s FR. The association between FFA and FR (*p*-value for interaction: *p* = 0.001) was inversed for girls and boys with effects of −0.376 (95% CI −0.522, −0.029) and 0.278 (0.055, 0.500) respectively.

### 3.5. Secondary Analysis

In essence, the same results were found when all ethnicities were included. While there were small differences in significance of the associations, all associations were in the same direction with only slight differences in effect sizes (see supplementary Tables S3 and S4).

## 4. Discussion

The aim of this study was to investigate the association between the maternal prenatal lipid profile assessed in early pregnancy and offspring’s eating behaviour and food intake at the age of five. TG was, according to expectations, positively associated with increased food responsiveness in the offspring. Contrarily to expectations, higher TC concentrations were associated with increased food avoidant behaviour, decreased food approaching behaviour and lower food intake in the offspring. ApoA1 and ApoB were positively associated with food avoidant behaviour, while increased ApoA1 concentrations were also associated with lower energy intake. This is the first evidence for associations between maternal lipid levels in early pregnancy and eating behaviour and food intake in children.

In our study, lower levels of TC, ApoA1 and ApoB concentrations were associated with adverse eating behaviour and also higher energy intake in case of TC and ApoA1 in the offspring at the age of five. While these results were unexpected, they could be explained by the high foetal cholesterol demands during pregnancy. Especially during early gestation demands are high, as foetal synthesis is not yet functional at that moment [37,39]. Furthermore, cholesterol plays an important role in the foetal development of cell membranes and the production of precursors of bile acids and steroid hormones [39,40]. Accordingly, low maternal cholesterol is associated with adverse foetal neurological development [40] and lower birthweight [41]. This is in line with our results showing that lower cholesterol concentrations are associated with adverse eating behaviour and increased food intake. 

We further found that TG was, as hypothesized, positively associated with food responsiveness in the offspring. This is in line with animal studies showing that increased perinatal TG concentrations play a role in the programming of hyperphagia [42], we are unaware of human studies on this topic and therefore could not compare our results to other findings. More research in humans is needed to confirm our results.

In line with Gademan’s research, which showed a positive association between maternal FFA and offspring’s fat percentage, BMI and obesity risk [22], we found a significant association between FFA and FR, with associations being only significant for boys. Males generally seem to be more susceptible to a disadvantageous foetal environment than females [43]. Males for instance, seem to be more sensitive to maternal high-fat diet and glucose dysregulation than females [44,45]. Replication in future studies is necessary before conclusions can be drawn, as this also might be an incidental finding.

When looking at the associations between the maternal lipid profile and eating behaviour in the offspring, we found relatively small effect sizes. However, even small differences in eating behaviour in young children might persist and it has been shown that increased food approaching behaviour becomes more pronounced, while food avoidant behaviour decreases when children grow older [46]. Moreover, when looking at estimated associations between TC, ApoA1 and food intake, effect sizes were much larger. For instance, an increase of maternal ApoA1 by 1 g/L was associated with a decrease of 185.99 kcal per day in the offspring. A study estimated that by decreasing daily energy intake by 100 kcal, weight gain could be prevented in the majority of the population [47]. A decrease of this magnitude in children might be even more effective and our findings are therefore most likely of clinical relevance.

A strength of this study is the large study sample, which increases the chance of finding significant results and decreases the influence of outliers on the results. The prospective design, wide range of outcome variables and measured covariables of our study reduce the risk of bias. Another strength is the long follow-up period which increases the clinical relevance of outcomes. The choice of excluding all non-Dutch participants is, one the one hand, a strength, as it increases homogeneity of the study sample and reduces bias. On the other hand, it can be seen as a limitation, as it reduces the generalizability of this study. However, including non-Dutch participants in a secondary analysis did not seem to affect our outcomes considerably.

Another limitation of this study is the possibility that selection bias might have occurred. Firstly, the study participants were healthier and higher educated than those in the non-response group. We therefore also expected lipid concentrations to be in a low, healthy range. Indeed, when comparing the lipid concentrations of all participants to reference values specified for the first two trimesters of pregnancy, only very few measurements were deviant [37]. For example, only two out of all participants had a TG concentration higher than the recommended maximum of 4.3 mmol/L [37]. The range of lipid concentrations is therefore limited, which might have led to an underestimation of effects in the general population.

In addition, as multiple variables and outcomes were analysed in this study, the issue of multiple testing arises, increasing the chance of a type 2 error. However, as this is an explorative study, we did not adjust *p*-values for multiple comparisons to insure not to miss any possible effects. Moreover, if we would have applied the Bonferroni correction and accordingly would have handled a *p*-value of *p* = 0.00142, still, several of the results would be significant.

A further limitation of this study might be the methods to assess the outcome variables. As CEBQ and FFQ are both parent-report questionnaires, parents’ perceptions about their own food intake and behaviour might influence results. For example, parents might wish to present their child in a good light and give socially desirable answers. However, both questionnaires have been validated in Dutch populations [25,36], indicating that both CEBQ and FFQ are reliable. Furthermore, CEBQ subscales were, in accordance to other research [9], highly correlated with the offspring’s BMI and energy intake, which indicates good internal validity. Lastly, adjustments for the range of confounders only slightly changed effect sizes, which indicates minimal residual confounding.

Another limitation of this study is that maternal lipid concentrations were only measured once and in a non-fasting state. Variation following from differences in dietary intake could not be taken into account. Furthermore, maternal energy and fat intake were not measured during pregnancy, therefore we could not check for associations between maternal food intake and blood lipids. However, previous research has indicated that fat intake during pregnancy is related to lipid levels in blood [21]. A final limitation is that the data analysed in this study were collected over ten years ago, which reduces the generalizability of our findings to our current time and newer studies will have to replicate our results.

## 5. Conclusions

In conclusion, we found that maternal prenatal TC, ApoA1 and ApoB were negatively associated with adverse eating behaviour and food intake in the offspring, while maternal TG concentrations were positively associated with food responsiveness in the offspring at the age of five. As this is the first performed in humans concerning this subject, it was exploratory in nature and therefore no implications for practice arise so far. However, this research adds to the amount of evidence pointing out the importance of nutrition during pregnancy and its effect on the health of future generations. More research is needed to further highlight the link between maternal diet and offspring’s vulnerability to develop obesity. Possible future studies should include women with a wider range in lipid concentrations to increase generalizability. Also, measuring food intake in the pregnant women in addition to lipid concentrations should be considered, to gain more insight into possible underlying pathways. Furthermore, future research should include a longer follow-up time to investigate possible effects on the long term. Ultimately, further research should lead to recommendations for public health improvements to target childhood obesity.

## Figures and Tables

**Table 1:** Maternal lipid concentrations according to maternal and offspring characteristics.

	N (%)	TC Mean (SD)	ApoA1 Mean (SD)	ApoB Mean (SD)	TG Mean (SD)	FFA Mean (SD)
**Maternal characteristics**		(mmol/L)	(g/L)	(g/L)	(mmol/L)	(mmol/L)
Age (years)						
<25	40 (2.8)	5.25 (0.85)	1.56 (0.20)	0.79 (0.16)	1.71 *** (0.74)	0.34 (0.20)
25–35 *(reference)*	1.122 (79.8)	5.19 (0.85)	1.62 (0.21)	0.74 (0.17)	**1.42 (0.49)**	**0.29 (0.21)**
>35	244 (17.4)	5.17 (0.81)	1.58 (0.19)	0.74 (0.15)	1.46 (0.49)	0.33 ** (0.19)
Parity						
0	839 (59.7)	5.24 ** (0.82)	1.64 *** (0.21)	0.75 (0.17)	1.44 (0.49)	0.29 (0.16)
≥1	567 (40.3)	**5.11 (0.87)**	**1.57 (0.20)**	0.74 (0.17)	1.43 (0.52)	0.31 (0.16)
Education after primary school						
0–5 years	71 (5.1)	5.44 ** (0.99)	1.60 (0.20)	0.80 ** (0.20)	1.63 *** **(0.68)**	0.35 ** (0.18)
6–10 years	472 (33.6)	5.26 * (0.85)	1.63 (0.21)	0.76 ** (0.17)	1.51 *** (0.56)	0.31 * (0.16)
>10 years *(reference)*	861 (61.3)	**5.13 (0.82)**	1.60 (0.21)	**0.73 (0.17)**	**1.38 (0.44)**	**0.29 (0.16)**
Smoking during pregnancy						
Yes	113 (8.0)	5.38 * (0.88)	1.57 (0.21)	0.81 *** (0.19)	1.68 *** (0.67)	0.32 (0.17)
No	1.293 (92.0)	**5.17 (0.84)**	1.61 (0.21)	**0.74 (0.17)**	**1.41 (0.48)**	0.30 (0.16)
Alcohol intake during pregnancy						
Yes	465 (33.1)	5.14 (5.14)	1.61 (0.21)	0.73 * (0.16)	1.37 ** (0.52)	0.29 (0.15)
No	940 (66.9)	5.21 (5.21)	1.61 (0.21)	**0.75 (0.17)**	**1.47 (0.46)**	0.30 (0.16)
pBMI (kg/m^2^)						
<18.5	40 (2.8)	4.99 (0.78)	1.57 (0.17)	0.72 (0.16)	1.42 (0.47)	0.32 (0.14)
18.5–24.9 *(reference)*	1.132 (80.5)	**5.14 (0.83)**	1.61 (0.21)	**0.73 (0.16)**	**1.41 (0.48)**	**0.29 (0.15)**
25–29.9	195 (13.9)	5.47 *** (0.90)	1.59 (0.21)	0.82 *** (0.17)	1.53 * (0.56)	0.33 * (0.18)
≥30	39 (2.8)	5.46 (0.72)	1.61 (0.19)	0.86 *** (0.18)	1.72 *** (0.52)	0.39 ** (0.26)
Weight gain since pregnancy						
Weight loss (>−3 kg)	174 (12.7)	5.23 (0.89)	1.60 (0.21)	0.77 * (0.19)	1.46 (0.48)	0.35 *** (0.20)
Weight stable (+/−3 kg) *(reference)*	832 (60.9)	5.16 (0.85)	1.61 (0.21)	**0.74 (0.17)**	1.42 (0.50)	**0.29 (0.14)**
Weight gain (>+3 kg)	360 (26.4)	5.21 (0.81)	1.61 (0.21)	0.75 (0.16)	1.45 (0.51)	0.30 (0.17)
Prenatal stress						
Low	385 (27.5)	5.18 (0.78)	1.60 (0.21)	0.75 (0.16)	1.47 (0.53)	0.30 (0.17)
High	1.017 (72.5)	5.19 (0.87)	1.61 (0.21)	0.74 (0.17)	1.42 (0.49)	0.30 (0.16)
**Child characteristics**						
Gender						
Boy	686 (48.8)	5.15 (0.82)	1.61 (0.21)	0.74 (0.17)	1.43 (0.47)	0.30 (0.16)
Girl	720 (51.2)	5.22 (0.86)	1.61 (0.20)	0.75 (0.17)	1.44 (0.53)	0.30 (0.16)
Birth weight						
≤2500 g	18 (1.3)	4.89 (0.70)	1.60 (0.21)	0.70 (0.18)	1.40 (0.55)	0.28 (0.15)
2500–4000 g *(reference)*	1.119 (79.7)	5.19 (0.84)	**1.62 (0.21)**	0.74 (0.17)	1.43 (0.50)	0.30 (0.16)
≥4000 g	267 (19.0)	5.21 (0.86)	1.57 ** (0.20)	0.76 (0.17)	1.45 (0.49)	0.29 (0.15)
Exclusive breastfeeding						
none	214 (15.2)	5.29 (0.84)	1.61 (0.20)	0.77 (0.17)	1.46 (0.48)	0.33 (0.19)
<1 month	68 (4.8)	5.17 (0.78)	1.60 (0.19)	0.75 (0.16)	1.46 (0.50)	0.30 (0.16)
1–2.9 months	374 (26.6)	5.20 (0.86)	1.62 (0.21)	0.74 (0.17)	1.40 (0.48)	0.31 (0.16)
3–5.9 months	487 (34.7)	5.13 (0.82)	1.61 (0.21)	0.73 (0.16)	1.46 (0.51)	0.29 (0.15)
≥6 months *(reference)*	262 (18.6)	5.20 (0.88)	1.59 (0.20)	0.75 (0.18)	1.43 (0.52)	0.30 (0.15)
Accelerated postnatal growth						
Yes	153 (14.0)	5.21 (0.80)	1.61 (0.21)	0.74 (0.17)	1.43 (0.50)	0.29 (0.15)
No	939 (86.0)	5.16 (0.84)	1.61 (0.20)	0.74 (0.17)	1.39 (0.43)	0.32 (0.19)
BMI at age 5						
Underweight	143 (13.3)	5.17 (0.81)	1.61 (0.22)	0.73 (0.17)	1.41 (0.50)	0.30 (0.16)
Normal weight *(reference)*	868 (80.6)	5.17 (0.86)	1.60 (0.21)	0.74 (0.17)	1.44 (0.49)	**0.30 (0.16)**
Overweight	66 (6.1)	5.39 (0.78)	1.61 (0.23)	0.79 (0.14)	1.42 (0.47)	0.35 * (0.21)

**Bold text** indicates a statistically significant *p*-value; * *p* < 0.05; ** *p* < 0.01; *** *p* < 0.001; Accelerated postnatal growth: Δ (SDS weight 6 months-SDS 1 month) ≥ 0.67. ApoA1: Apolipoprotein A1 (g/L); ApoB Apolipoprotein B (g/L); BMI age 5: Body Mass Index age 5, categories according to gender-specific Dutch references. FFA: Free fatty acids (mmol/L); pBMI: pre-pregnancy Body Mass Index; Prenatal anxiety (STAI score ≥ 43); TC: Total cholesterol (mmol/L); TG: Triglycerides (mmol/L); weight gain since pregnancy: Δ maternal weight year 5–pre-pregnancy weight. All lipids interpolated to 90 days gestational age.

**Table 2:** Mean values outcome measures.

	Mean (SD)	IQR
Enjoyment of food	2.56 (0.47)	2.20–2.80
Food responsiveness	1.88 (0.49)	1.50–2.25
Satiety responsiveness	2.32 (0.49)	2.00–2.60
Slowness of eating	2.41 (0.57)	2.00–2.75
Energy intake (kcal/day)	1507.99 (295.38)	1314.84–1679.89
Carbohydrate intake (g/day)	192.22 (39.07)	167.31–213.54
Fat intake (g/day)	52.12 (13.01)	43.20–59.05

**Table 3:** Correlations between offspring’s food intake, eating behaviour and BMI.

	EF	FR	SR	SE	BMI (kg/m^2^)
Enjoyment of food	-----	0.467 ***	−0.553 ***	−0.510 ***	0.277 ***
Food responsiveness	0.467 ***	-----	−0.115 ***	−0.125 ***	0.294 ***
Satiety responsiveness	−0.553 ***	−0.115 ***	-----	0.637 ***	−0.261 ***
Slowness of eating	−0.510 ***	−0.125 ***	0.637 ***	-----	−0.288 ***
Kcal intake (kcal/day)	0.178 ***	0.122 ***	−0.153 ***	−0.146 ***	0.047
Fat intake (g/day)	0.145 ***	0.074 *	−0.113 ***	−0.085 **	0.011
Carbohydrate intake (g/day)	0.140 ***	0.111 ***	−0.129 ***	−0.147 ***	0.044

**Bold text** indicates a statistically significant *p*-value; * *p* < 0.05; ** *p* < 0.01; *** *p* < 0.001; BMI: Body Mass Index offspring age 5; EF: Enjoyment of food; FR: Food responsiveness; SE: Slowness of eating; SR: Satiety responsiveness.

**Table 4:** Association between prenatal maternal lipid profile and offspring’s eating behaviour at age 5.

	Enjoyment of Food	Food Responsiveness	Satiety Responsiveness	Slowness of Eating
	β (95% CI)	β (95% CI)	β (95% CI)	β (95% CI)
TC (mmol/L)				
Crude	−0.033 * (−0.062, −0.003)	0.010 (−0.021, 0.041)	0.059 *** (0.029, 0.089)	0.050 ** (0.015, 0.085)
Adjusted ^1^	−0.037 * (−0.067, −0.007)	0.000 (−0.031, 0.032)	0.052 *** (0.021, 0.083)	0.048 ** (0.012, 0.084)
ApoA1 (g/L)				
Crude	−0.119 (−0.239, 0.001)	−0.045 (−0.171, 0.081)	0.099 (−0.025, 0.222)	0.154 * (0.009, 0.299)
Adjusted ^1^	−0.128 * (−0.250, −0.005)	−0.039 (−0.166, 0.088)	0.119 (−0.006, 0.244)	0.162 * (0.014, 0.309)
ApoB (g/L)				
Crude	−0.116 (−0.264, 0.031)	0.063 (−0.091, 0.217)	0.236 ** (0.085, 0.387)	0.202 * (0.024, 0.380)
Adjusted ^1^	−0.131 (−0.285, 0.023)	0.008 (−0.152, 0.168)	0.185 * (0.027, 0.342)	0.196 * (0.011, 0.382)
TG (mmol/L)				
Crude	0.040 (−0.009, 0.090)	0.086 ** (0.034, 0.137)	0.024 (−0.027, 0.076)	0.000 (−0.060, 0.059)
Adjusted ^1^	0.048 (−0.003, 0.099)	0.076 ** (0.023, 0.129)	0.000 (−0.052, 0.053)	−0.012 (−0.073, 0.050)
FFA (mmol/L)				
Crude	0.001 (−0.156, 0.158)	0.048 (−0.116, 0.212)	0.141 (−0.020, 0.302)	0.055 (−0.134, 0.244)
Adjusted ^1^	−0.007 (−0.167, 0.153)	−0.007 (−0.173, 0.160)	0.107 (−0.057, 0.270)	0.051 (−0.142, 0.243)

**Bold text** indicates a statistically significant *p*-value; * *p* < 0.05; ** *p* < 0.01; *** *p* < 0.001.; ^1^ Adjusted for: educational status, pre-pregnancy body mass index, parity, prenatal maternal anxiety, offspring’s gender, duration exclusive breastfeeding, increased infant growth, maternal weight gain since pregnancy; ApoA1: Apolipoprotein A1 (g/L); ApoB Apolipoprotein B (g/L); FFA: Free fatty acids (mmol/L); TC: Total cholesterol (mmol/L); TG: Triglycerides (mmol/L). All lipids interpolated for mean gestational age at blood sampling (90 days).

**Table 5:** Association between prenatal maternal lipid profile and offspring’s food intake at age 5.

	Kcal Intake (kcal/day)	Fat Intake (g/day)	Carbohydrate Intake (g/day)
	β (95% CI)	β (95% CI)	β (95% CI)
TC (mmol/L)			
Crude	−32.63 ** (−54.88, −10.39)	−1.57 ** (−2.55, −0.59)	−3.25 * (−6.19, −0.30)
Adjusted ^1^	−26.33 * (−48.42, −4.24)	−1.38 ** (−2.36, −0.40)	−2.27 (−5.21, 0.68)
ApoA1 (g/L)			
Crude	−183.62 *** (−270.70, −96.54)	−6.59 *** (−10.43, −2.76)	−23.74 *** (−35.37, −12.12)
Adjusted ^1^	−185.99 *** (−271.87, −100.10)	−6.86 *** (−10.66, −3.06)	−23.55 *** (−35.11, −11.99)
ApoB (g/L)			
Crude	−87.31 (−197.45, 22.82)	−4.80 (−9.63, 0.04)	−7.85 (−22.55, 6.85)
Adjusted ^1^	−57.00 (−168.33, 54.33)	−4.09 (−8.99, 0.82)	−2.68 (−17.67, 12.30)
TG (mmol/L)			
Crude	−2.06 (−39.69, 35.57)	−0.46 (−1.39, 0.48)	0.89 (−4.08, 5.86)
Adjusted ^1^	1.59 (−36.02, 39.21)	−0.39 (−2.06, 1.27)	1.51 (−3.50, 6.51)
FFA (mmol/L)			
Crude	−89.00 (−203.26, 25.26)	−1.95 (−6.97, 3.08)	−15.02 (−30.25, 0.21)
Adjusted ^1^	−74.40 (−186.76, 37.95)	−1.31 (−6.27, 3.64)	−13.42 (−28.52, 1.69)

**Bold text** indicates a statistically significant *p*-value; * *p* < 0.05; ** *p* < 0.01; *** *p* < 0.001; ^1^ Adjusted for: educational status, pre-pregnancy body mass index, parity, prenatal maternal anxiety, offspring’s gender, duration exclusive breastfeeding, increased infant growth, maternal weight gain since pregnancy; ApoA1: Apolipoprotein A1 (g/L); ApoB Apolipoprotein B (g/L); FFA: Free fatty acids (mmol/L); TC: Total cholesterol (mmol/L); TG: Triglycerides (mmol/L). All lipids interpolated for mean gestational age at blood sampling (90 days).

## Supplementary Materials

The following are available online at http://www.mdpi.com/2072-6643/10/8/1026/s1, Table S1: Non-response analysis, Table S2: CEBQ scores according to maternal and offspring characteristics, Table S3: Secondary analysis; Association between prenatal maternal lipid profile and offspring’s eating behaviour at age 5, Table S4: Secondary analysis; Association between prenatal maternal lipid profile and offspring’s food intake at age 5.
