# Supplementary material for: Maternal Lipid Concentrations during Early Pregnancy and Eating Behaviour and Energy Intake in the Offspring
Source: Nutrients. 2018 Aug 6;10(8):1026. doi: 10.3390/nu10081026 (PMC6115740; doi:10.3390/nu10081026)
Supplement: Supplementary file 1 [file nutrients-10-01026-s001.pdf]

Supplemental table S1. Non-response analysis

	Included (%)	Excluded (%)	p-value
	(n=1463)	(n=672)	
Age (years)			
<25	**41 (2.8)**	**52 (7.7)**	**<0.001**
25-35	**1171 (80.0)**	**530 (78.9)**	
>35	**251 (17.2)**	**90 (13.4)**	
Parity			
0	878 (60.0)	404 (60.1)	0.963
≥ 1	585 (40.0)	268 (39.9)	
Education after primary school			
0-5 years	**72 (4.9)**	**72 (10.7)**	**<0.001**
6-10 years	**490 (33.5)**	**276 (41.1)**	
> 10 years	**899 (61.5)**	**323 (48.1)**	
Smoking during pregnancy			
yes	119 (8.1)	66 (9.8)	0.198
no	1344 (91.9)	606 (90.2)	
Alcohol intake during pregnancy			
yes	**489 (33.4)**	**187 (27.8)**	**0.010**
no	**973 (66.6)**	**485 (72.2)**	
Pre-pregnancy BMI (kg/m2)			
< 18.5	42 (2.9)	31 (4.6)	0.223
18.5-24.9	1178 (80.5)	533 (79.3)	
25-29.9	201 (13.7)	91 (13.5)	
≥ 30	42 (2.9)	17 (2.5)	

Supplemental table S2. CEBQ scores according to maternal and offspring characteristics

	n (%)	EF (SD)	FR (SD)	SR (SD)	SE (SD)
**Maternal characteristics**					
Age (years)					
<25	41 (2.8)	2.67 (0.49)	**2.12** ** **(0.65)**	2.36 (0.52)	2.46 (0.67)
25-35 *(reference)*	1170 (80.0)	2.55 (0.48)	**1.87 (0.49)**	2.32 (0.49)	2.41 (0.58)
>35	251 (17.2)	2.58 (0.46)	1.91 (0.46)	2.28 (0.46)	2.38 (0.51)
Parity					
0	877 (60.0)	2.57 (0.47)	1.87 (0.49)	2.30 (0.48)	2.41 (0.57)
≥ 1	585 (40.0)	2.56 (0.48)	1.90 (0.50)	2.34 (0.49)	2.41 (0.56)
Education after primary school					
0-5 years	72 (4.9)	2.59 (0.51)	**2.03** * **(0.66)**	2.38 (0.56)	2.45 (0.62)
6-10 years	489 (33.5)	2.54 (0.48)	1.87 (0.50)	2.32 (0.50)	2.42 (0.52)
> 10 years *(reference)*	899 (61.6)	2.57 (0.47)	**1.88 (0.48)**	2.31 (0.47)	2.40 (0.59)
Smoking during pregnancy					
Yes	119 (8.1)	2.51 (0.45)	1.89 (0.51)	2.40 (0.50)	2.47 (0.56)
No	1343 (91.9)	2.57 (0.48)	1.88 (0.49)	2.31 (0.48)	2.40 (0.57)
Alcohol intake during pregnancy					
Yes	489 (33.5)	2.55 (0.50)	1.90 (0.49)	2.31 (0.50)	2.42 (0.58)
No	972 (66.5)	2.57 (0.46)	1.87 (0.50)	2.32 (0.48)	2.40 (0.56)
pBMI (kg/m2)					
< 18.5	42 (2.9)	**2.36** * **(0.54)**	**1.60** ** **(0.42)**	2.32 (0.54)	2.60 (0.61)
18.5-24.9 *(reference)*	1178 (80.6)	**2.57 (0.48)**	**1.89 (0.49)**	**2.31 (0.49)**	2.41 (0.57)
25-29.9	201 (13.7)	2.59 (0.43)	1.89 (0.48)	2.32 (0.46)	2.37 (0.57)
≥ 30	41 (2.8)	2.54 (0.45)	1.99 (0.62)	**2.54** * **(0.52)**	2.52 (0.45)
Weight gain since pregnancy					
Weight loss (> -3kg)	182 (15.4)	2.60 (0.47)	1.94 (0.57)	2.31 (0.49)	2.39 (0.58)
Weight stable (+/-3kg) *(reference)*	864 (73.2)	2.56 (0.47)	1.87 (0.49)	2.30 (0.49)	2.39 (0.57)
Weight gain (> +3kg)	374 (31.7)	2.54 (0.47)	1.88 (0.47)	2.35 (0.47)	2.46 (0.55)
Prenatal stress					
Low	406 (27.8)	2.56 (0.49)	**1.93** * **(0.51)**	2.34 (0.48)	2.41 (0.55)
High	1052 (72.2)	2.56 (0.47)	**1.86 (0.49)**	2.31 (0.49)	2.41 (0.58)
**Child characteristics**					
Gender					
Boy	711 (48.6)	2.57 (0.46)	1.87 (0.47)	**2.28** ** **(0.47)**	**2.36** ** **(0.55)**
Girl	751 (51.4)	2.55 (0.48)	1.90 (0.52)	**2.35 (0.49)**	**2.45 (0.58)**
Birth weight					
<=2500g	18 (1.2)	2.53 (0.58)	1.96 (0.61)	2.52 (0.66)	2.50 (0.51)
2500-4000g *(reference)*	1166 (79.9)	**2.54 (0.47)**	1.87 (0.49)	**2.33 (0.49)**	**2.43 (0.57)**
≥ 4000g	276 (18.9)	**2.64** ** **(0.46)**	1.93 (0.52)	**2.25** * **(0.47)**	**2.31** ** **(0.56)**
Exclusive breastfeeding					
None	223 (15.3)	2.58 (0.48)	1.88 (0.51)	2.32 (0.52)	2.41 (0.57)
< 1 month	69 (4.7)	2.53 (0.51)	1.84 (0.56)	2.34 (0.52)	2.45 (0.59)
1-2.9 months	389 (26.6)	2.57 (0.47)	1.93 (0.49)	2.32 (0.45)	2.42 (0.56)
3-5.9 months	509 (34.8)	2.55 (0.47)	1.87 (0.48)	2.31 (0.48)	2.41 (0.57)
≥ 6 months *(reference)*	271 (18.5)	2.56 (0.46)	1.84 (0.50)	2.31 (0.50)	2.39 (0.58)
Accelerated postnatal growth					
Yes	159 (14.0)	2.62 (0.47)	**1.96** * **(0.49)**	2.28 (0.50)	2.37 (0.60)
No	976 (86.0)	2.55 (0.47)	**1.86 (0.48)**	2.33 (0.49)	2.43 (0.55)

* p <0.05 ** p <0.01 *** p <0.001

Accelerated postnatal growth: Δ (SDS weight 6 months - SDS 1 month) ≥ 0.67; EF: Enjoyment of food; FR: Food responsiveness; pBMI: Pre-pregnancy Body mass index; Prenatal stress: measured as general anxiety (STAI score ≥ 43); SE: Slowness of eating; SR: Satiety responsiveness; Weight gain since pregnancy: Δ Maternal weight year 5 - pre-pregnancy weight.

Supplemental Table S3. Secondary analysis; Association between prenatal maternal lipid profile and offspring's eating behavior at age 5, all ethnicities included (N=2044)

		Enjoyment of Food	Food Responsiveness	Satiety Responsiveness	Slowness of Eating
		β (95%CI)	β (95%CI)	β (95%CI)	β (95%CI)
TC (mmol/L)	Crude	**-0.034* (-0.060, -0.008)**	-0.001 (-0.028, 0.025)	**0.040** (0.013, 0.066)**	**0.052*** (0.021, 0.082)**
	Adjusted[1]	**-0.038** (-0.065, -0.012)**	-0.007 (-0.034, 0.020)	**0.040** (0.013, 0.066)**	**0.051** (0.020, 0.081)**
ApoA1 (g/L)	Crude	-0.085 (-0.185, 0.016)	-0.061 (-0.163, 0.041)	0.048 (-0.054, 0.151)	0.102 (-0.015, 0.219)
	Adjusted[1]	**-0.104* (-0.206, -0.002)**	-0.061 (-0.165, 0.042)	0.084 (-0.019, 0.187)	0.110 (-0.008, 0.228)
ApoB (g/L)	Crude	**-0.144* (-0.275, -0.013)**	0.022 (-0.111, 0.156)	**0.185** (0.052, 0.318)**	**0.224** (0.071, 0.376)**
	Adjusted[1]	**-0.137* (-0.271, -0.002)**	-0.006 (-0.143, 0.131)	0.129 (-0.007, 0.265)	**0.202* (0.045, 0.358)**
TG (mmol/L)	Crude	**0.043* (0.002, 0.085)**	**0.071** (0.029, 0.113)**	-0.008 (-0.051, 0.035)	0.009 (-0.039, 0.057)
	Adjusted[1]	**0.056* (0.013, 0.098)**	**0.068** (0.024, 0.111)**	-0.032 (-0.075, 0.011)	-0.003 (-0.052, 0.047)
FFA (mmol/L)	Crude	0.013 (-0.120, 0.146)	0.071 (-0.064, 0.207)	**0.144* (0.009, 0.279)**	0.099 (-0.056, 0.253)
	Adjusted[1]	0.042 (-0.094, 0.178)	0.046 (-0.091, 0.184)	0.074 (-0.062, 0.211)	0.063 (-0.093, 0.218)

* p <0.05 ** p <0.01 *** p <0.001

ApoA1: Apolipoprotein A1 (g/L); ApoB Apolipoprotein B (g/L); FFA: Free fatty acids (mmol/L); TC: Total cholesterol (mmol/L); TG: Triglycerides (mmol/L). All lipids interpolated for mean gestational age at blood sampling (90 days).

Adjusted for: ethnicity, educational status, pre-pregnancy Body Mass Index, parity, prenatal maternal stress, offspring's gender, duration exclusive breastfeeding, increased infant growth, maternal weight gain since pregnancy.

Supplemental table S4. Secondary analysis; Association between prenatal maternal lipid profile and offspring's food intake at age 5 including all ethnicities (N=2044)

		Kcal intake (kcal/d)	Fat intake (g/d)	Carbohydrate intake (g/d)
		β (95%CI)	β (95%CI)	β (95%CI)
TC (mmol/L)	crude	**-31.30** (-53.06, -9.55)**	**-1.16* (-2.10, -0.23)**	**-4.06** (-6.92, -1.19)**
	adjusted[1]	**-26.05* (-47.64, -4.45)**	**-1.01* (-1.94, -0.07)**	**-3.28* (-6.14, -0.42)**
ApoA1 (g/L)	crude	**-120.48** (-203.63, -37.33)**	-3.13 (-6.70, 0.44)	**-17.81** (-28.80, -6.83)**
	adjusted[1]	**-99.02* (-181.20, -16.84)**	-2.20 (-5.73, 1.34)	**-15.67** (-26.58, -4.76)**
ApoB (g/L)	crude	-45.20 (-154.04, 63.64)	-2.52 (-7.18, 2.14)	-5.37 (-19.75, 9.02)
	adjusted[1]	-52.65 (-162.58, 57.27)	-3.29 (-8.01, 1.43)	-5.03 (-19.63, 9.58)
TG (mmol/L)	crude	28.06 (-6.68, 62.81)	0.86 (-0.64, 2.35)	4.10 (-0.48, 8.68)
	adjusted[1]	15.64 (-19.50, 50.79)	0.30 (-1.22, 1.82)	2.77 (-1.88, 7.41)
FFA (mmol/L)	crude	40.97 (-68.18, 150.12)	2.53 (-2.15, 7.20)	2.47 (-11.96, 16.90)
	adjusted[1]	11.54 (-97.02, 120.10)	1.38 (-3.28, 6.04)	-1.18 (-15.60, 13.24)

* $p < 0.05$ ** $p < 0.01$ *** $p < 0.001$

Adjusted for: ethnicity, educational status, pregravid Body Mass Index, parity, prenatal maternal stress, offspring's gender, duration exclusive breastfeeding, increased infant growth, maternal weight gain since pregnancy.

ApoA1: Apolipoprotein A1 (g/L); ApoB Apolipoprotein B (g/L); FFA: Free fatty acids (mmol/L); TC: Total cholesterol (mmol/L); TG: Triglycerides (mmol/L). All lipids interpolated for mean gestational age at blood sampling (90 days).
